# Review of cocaine-induced brain vascular and cellular function changes measured *in vivo* with optical imaging

**DOI:** 10.1117/1.NPh.12.S1.S14611

**Published:** 2025-05-28

**Authors:** Congwu Du, Hyomin Jeong, Alan P. Koretsky, Yingtian Pan

**Affiliations:** a Stony Brook University, Department of Biomedical Engineering, Stony Brook, New York, United States; b National Institute of Neurological Disorders and Stroke, National Institutes of Health, Bethesda, Maryland, United States

**Keywords:** optical imaging *in vivo*, neuroimaging, cerebral blood flow, hemoglobin oxygenation and deoxygenation, cocaine, addiction

## Abstract

**Significance:**

Cocaine exerts effects on vascular and cellular functions in the brain. The interactions among cerebrovasculature, neurons, and astrocytes and their dynamic changes during exposure complicate the understanding of its effects. Therefore, there is a need for simultaneous, multiparameter *in vivo* measurements to accurately distinguish these effects.

**Aim:**

A multimodal optical imaging approach that is tailored to investigate cocaine’s effects on cerebrovasculature, neurons, and astrocytes in high-spatiotemporal resolution and large field of view is presented with comparisons to other modalities.

**Approach:**

This approach integrates optical coherence tomography, fluorescence, and spectral absorption imaging to permit high-resolution imaging of 3D cerebrovessels, cerebral blood flow (CBF), changes in oxygenated/deoxygenated hemoglobin, and large-scale cellular activities via intracellular calcium fluorescence expressed through genetically encoded calcium indicators in the mouse cortex.

**Results:**

Results show that cocaine induces vasoconstriction and reduces CBF, thus increasing the susceptibility of the brain to ischemia with chronic exposure. Moreover, cocaine alters neuronal activity and frontal responses to deep brain stimulation.

**Conclusions:**

These findings on cocaine’s effects on the neuro-astroglial-vascular network in the prefrontal cortex highlight the unique capacity of optical imaging to reveal the cellular and vascular mechanisms underlying cocaine’s neurotoxic effects on brain function.

## 1 Introduction

### 1.1 Importance of Imaging Cellular and Vascular Effects of Cocaine on the Brain

Cocaine exerts a direct influence on cerebral blood vessels and neuronal function in the brain. This impact extends to glial cells, particularly astrocytes, which play a key role in neurovascular coupling—a process that regulates cerebral blood flow (CBF) in accordance with fluctuations in neuronal activity. Neurovascular coupling can be disrupted by brain diseases including cocaine addiction.^1^ For example, brain imaging studies have documented the remarkable decrease in CBF in cocaine users.^2^^–^^5^ From animal studies, chronic cocaine has been shown to trigger marked vasoconstriction and CBF decreases^6^^–^^8^ including transient ischemic attacks in parallel to associated behavioral deficits (e.g., paralysis).^9^ Meanwhile, acute and chronic cocaine depresses neuronal activity.^10^^–^^13^ In addition, in clinical studies, regional CBF decline was observed in the brain of cocaine users compared with that of non-users,^5^ and this finding has been replicated in multiple studies (summarized in Clare et al.^14^) to collectively support the linkage between cocaine use and CBF abnormalities in the brain. In addition, functional connectivity was shown to be dysfunctional in patients with cocaine-use disorders using functional magnetic resonance imaging (fMRI).^15^ Significant alterations were observed in connectivity among the prefrontal cortex (PFC), nucleus accumbens (NAc), and ventral tegmental area (VTA), regions that are brain reward circles associated with addiction.^16^^,^^17^ Other optical modalities such as functional near-infrared spectroscopy (fNIRS) have also been used for human studies, reporting increased neuronal activity in the PFC associated with cocaine use during decision-making tasks.^18^ However, the mechanisms underlying these compound effects of cocaine on neurons and vessels remain poorly understood. These effects may arise from (1) *vascular effects*—direct vasoconstrictive properties of cocaine and/or indirect, secondary vasoconstriction mediated by the release of sympathomimetic amines;^19^ (2) *neuronal deficits*—indirect consequence of diminished neural activity and metabolic demand;^5^ and/or (3) *astrocyte dysfunction*—which results in impaired mediation of synaptic transmission, neuroplasticity, extrasynaptic glutamate, and CBF homeostasis.^20^^–^^22^ However, addressing these gaps in knowledge has been challenging, largely due to the limited spatiotemporal resolution of current imaging techniques that hinder the simultaneous assessment of cocaine’s vascular and neuronal effects. Consequently, effectively separating the cellular from the vascular contributions of drugs such as cocaine remains a technical challenge.

### 1.2 Technical Challenges to Image Neuro-Astroglial-Vascular (NAV) Function *In vivo* for Cocaine Studies

The interactions among vascular systems, neurons, and astrocytes, along with the temporal dynamics of cocaine exposure, complicate the understanding of the impact of cocaine on the brain. Therefore, there is a need for simultaneous, multiparameter *in vivo* measurements to accurately distinguish these effects. Neuroimaging techniques such as positron emission tomography (PET),^5^^,^^23^^,^^24^ fMRI,^25^^,^^26^ and diffuse optical imaging (e.g., fNIRs^27^^–^^29^) have significantly advanced our ability to investigate brain function, including neuronal activity and the responses of cerebral vessels that supply the oxygen necessary for metabolic processes.^30^ However, these macroscopic imaging modalities are largely constrained by their limited spatiotemporal resolution, making it challenging to resolve individual vascular compartments or cells in the living brain. Concurrently, in combination with genetically encoded calcium indicators (GECIs), *in vivo* ${\mathrm{Ca}}^{2+}$ fluorescence imaging^31^^–^^33^ enables the detection of neural activity from the brain of a behaving animal,^34^ which can be used to identify the neuronal basis of reward-related behavior.^35^^–^^38^ In addition, with advances in *in vivo* imaging techniques such as confocal or two-photon microscopies, astrocyte activity can be detected in living brains along with neuronal calcium.^39^^,^^40^ Yet, these methods are hindered by small fields of view (FOV) and require a fluorescence contrast agent to detect CBF, which compromises the ability to track persistent CBF changes and long-term effects. Furthermore, the limited FOV and imaging depth for ${\mathrm{Ca}}^{2+}$ fluorescence imaging^41^^,^^42^ may prevent these optical microscopy techniques from studying changes in deep cortical layers even in the mouse, including important areas for cocaine addiction such as the medial frontal cortex (mPFC).

To tackle this problem, we have developed multimodality optical neuroimaging, referred to as a multimodal imaging platform (MIP).^43^ This system includes multiwavelength imaging (MWI) and laser speckle contrast imaging (LSCI)^44^ that enable simultaneous detection of cocaine-induced CBF, cerebral blood volume (CBV), and hemoglobin oxygenation (${\mathrm{HbO}}_{2}$) changes in brain tissue at high spatiotemporal resolutions and large FOV.^43^ This system can be co-registered with digital-frequency-ramping optical coherence tomography (DFR-OCT^45^) to enable *in vivo* quantitative 3D imaging of CBF networks to the level of capillaries.^8^^,^^43^^,^^44^ In addition, the fluorescence microscopy in the MIP allows us to image cocaine-induced neuronal and astrocyte changes by detecting intracellular calcium signaling.^46^^,^^47^ These technological accomplishments have laid a solid foundation to form the multimodality simultaneous imaging platform (MIP).^43^^,^^47^ This approach has been applied for simultaneous imaging of neuronal and cortical vascular changes that occur after acute and chronic cocaine administration.^6^^,^^7^^,^^10^^,^^11^^,^^14^^,^^48^^–^^50^ Most recently, a fl-ODM imaging platform that contains a custom hybrid dual-channel fluorescence imaging together with an ultrahigh-resolution optical coherence Doppler microscope (ODM) has been developed, which permits simultaneous images of neuronal-astroglial-vascular network from a living brain.^47^ We herein summarize these advanced imaging techniques and demonstrate their capability in investigating the pharmacological effects of cocaine on various aspects of brain function. These include the assessment of vascularization, hemodynamic responses, hemoglobin oxygenation, and neuronal and astrocytic activities in the cortical regions of experimental animal models.

## 2 Multimodality Optical Imaging and Technical Advances to Image a Living Brain

This section outlines some of the progress we have made in addressing the challenges in detecting cocaine-induced physiological changes in the cortical brain through these technological developments.

### 2.1 Simultaneous Imaging to Detect Hemodynamics, Tissue Oxygenation, and Cellular Activity

#### 2.1.1 Simultaneous imaging of CBF, CBV, and tissue hemoglobin oxygenation (${\mathrm{HbO}}_{2}$) changes

**Fig. 1:**
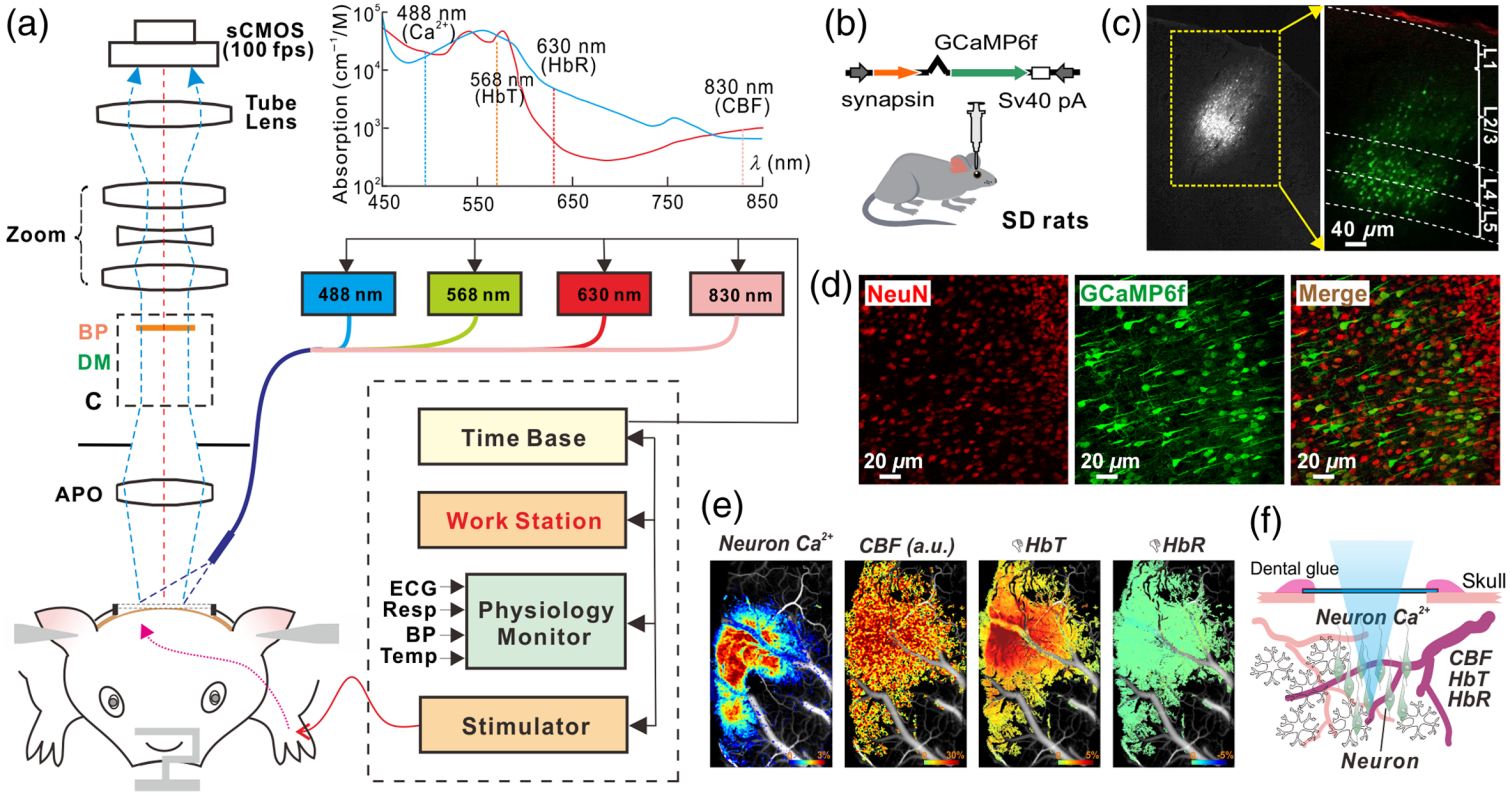
(a) Schematic illustrating the principle of our multimodality imaging platform (MIP) that integrates ${\mathrm{Ca}}^{2+}$ fluorescence imaging ($\lambda_{\mathrm{EX}1}=488\;\mathrm{nm}$) with a multiwavelength imager (MWI, $\lambda_{2}=568\;\mathrm{nm}$, and $\lambda_{3}=630\;\mathrm{nm}$) and laser speckle contrast imaging (LSCI, $\lambda_{4}=830\;\mathrm{nm}$). This system enables simultaneous detection of neuronal $Ca^{2+}$ activity with cerebral metabolic and hemodynamic changes. Sensory stimulation, such as electrical forepaw stimulation, is synchronized with the imaging platform through a shared time base, ensuring precise temporal alignment. (b) Viral injection to express a genetically encoded calcium indicator, GCaMP6f, to neurons in the somatosensory cortex. (c) *Ex vivo* fluorescence images to show the GCaMP6f distributions in cortical layers IV–V in the somatosensory cortex. (d) Confirmation via double staining with NeuN antibody that labels neurons and GFP antibody that labels GCaMP6f to show that all the GFP+ cells were neurons. (e) Simultaneous imaging of neuronal ${{\mathrm{Ca}}^{2+}}_{N}$, CBF, HbT, and HbR responses to forepaw electrical stimulation (image size: $3\times 5\;{\mathrm{mm}}^{2}$). (f) Schematic representation of simultaneous imaging of synchronized neuronal $Ca^{2+}$ dynamics within a neuronal population and the corresponding local hemodynamic responses. (Updated from Chen et al.^51^)

Figure 1 illustrates the MIP that has integrated multiwavelength spectroscopic imaging (MWI) with LSCI, enabling simultaneous monitoring of changes in CBF, CBV, or total hemoglobin concentration (HBT), oxygenated-([${\mathrm{HbO}}_{2}$]), and deoxygenated-hemoglobin ([HbR]) at a high spatiotemporal resolution.^43^^,^^51^ As shown in Fig. 1(a), the wavelengths of 568 and 630 nm were selected to distinguish changes in ${\mathrm{HbO}}_{2}$ and HbR from the cortex.^52^^,^^53^ In addition, LSCI ($\lambda_{4}=830\;\mathrm{nm}$) can provide the concurrent assessment of cocaine’s effects on the neurovascular network, including cocaine-induced changes in vascular CBF. Together with MWI, it can detect cocaine’s effects on hemodynamics (e.g., CBF, HbT) and tissue oxygenation metabolism (as indicated by ${\mathrm{HbO}}_{2}$ and HbR) in the living brain.

#### 2.1.2 Simultaneous neuronal ${{\mathrm{Ca}}^{2+}}_{N}$ *fluorescence and hemodynamic imaging to map brain activity*

Tracking brain activity through intracellular free ${\mathrm{Ca}}^{2+}$ measurement is a highly effective method, as the ${\mathrm{Ca}}^{2+}$ transients play a critical role in various neural processes including synaptic plasticity, neurotoxicity, and cell death (apoptosis). Traditionally, detecting neuronal ${{\mathrm{Ca}}^{2+}}_{N}$ changes required the use of fluorescent indicators for labeling. However, recent advancements allow for the use of genetically encoded neuron-specific ${\mathrm{Ca}}^{2+}$ indicators, such as AAV1.Syn.GCaMP6f.WPRE.SV40 (Penn Vector P2822)^54^ that are delivered via viral vectors. This approach now enables the detection of ${\mathrm{Ca}}^{2+}$ transients in neurons with a minimally invasive viral injection,^54^ making it possible to simultaneously measure cerebral hemodynamics in response to stimulation (i.e., forepaw or hind paw stimulation). This capability supports real-time mapping of regional brain activation *in vivo*, further advancing our understanding of neural functions and responses from a living brain.^51^^,^^55^^,^^56^

Figure 1(e) shows the overlapped spatial patterns of neuronal ${{\mathrm{Ca}}^{2+}}_{N}$ and hemodynamic responses (CBF, HbT, and HbR) following forepaw stimulation to highlight the ability of optical imaging to concurrently track both neuronal and vascular changes in real time during brain activation [Figs. 1(e) and 1(f)]. In addition, neuronal GCaMP6f expression shown in the *ex vivo* evidence [Figs. 1(c)–1(d)] confirms that the strong GCaMP ${{\mathrm{Ca}}^{2+}}_{N}$ signals detected with the optical imaging originate from cortical layers IV–V. More broadly, this technique offers a unique advantage in distinguishing between the neuronal and vascular effects of cocaine, a distinction that other brain imaging modalities are unable to achieve.

### 2.2 3D Quantitative Cerebral Blood Flow and Cerebrovascular Angiography Imaging

**Fig. 2:**
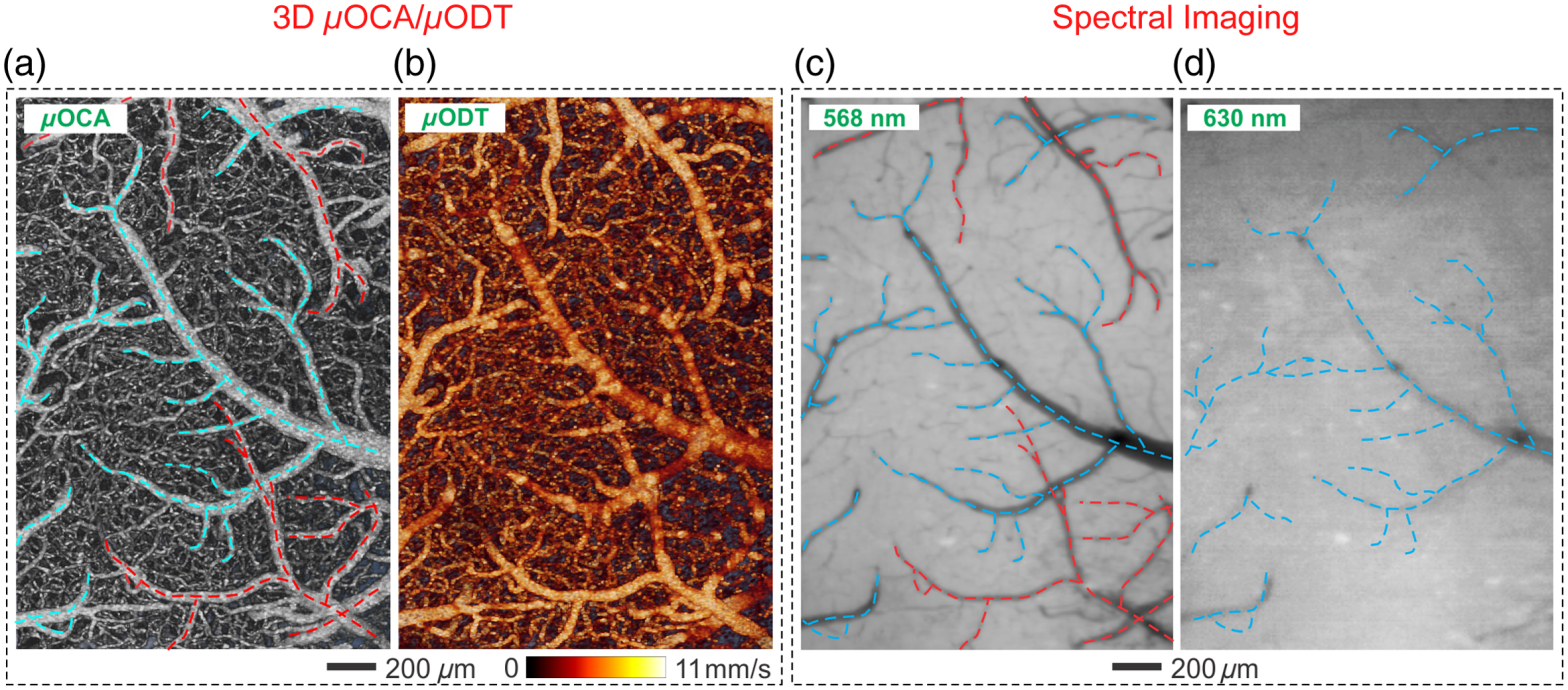
Multimodal imaging platform with μOCT for imaging cortical vascular network*in vivo*. (a) Ultra-high resolution optical coherence angiography (μOCA) of the vascular network from a mouse cortex *in vivo*. (b) Its quantitative map of CBF velocity obtained using ultra-high resolution optical coherence Doppler tomography (μODT) at a wavelength of $\lambda_{1}=1300\;\mathrm{nm}$. (c), (d) Spectral images of the cortex at $\lambda_{2}=568\;\mathrm{nm}$ and $\lambda_{3}=630\;\mathrm{nm}$, highlighting arteries (indicated by red arrows) and veins (indicated by blue arrows). Modified from Du et al.^59^

By integrating optical coherence tomography (OCT) with MWI, the MIP platform provides 3D quantitative images of CBF networks and cerebrovascular angiography. In other words, advances in OCT technology^57^ now allow *in vivo* visualization of both vascular angiography^58^ and CBF networks. Techniques such as phase-intensity-multiplexing ultrahigh-resolution optical Doppler tomography (ODT)^45^ and contrast-enhanced μODT^8^ have significantly increased the sensitivity of blood flow detection. These improvements enable fast, quantitative 3D CBF imaging for large and small vessels, including capillaries (e.g., ϕ<5  μm vessels with a flow rate of <20  μm/s) over a FOV of $3\times 3\times 1.5\;{\mathrm{mm}}^{3}$. Simultaneous imaging with ultrahigh-resolution optical coherence angiography (μOCA) and μODT further expands this capability. Figures 2(a)–2(b) show the neurovascular angiography and quantitative CBF velocity maps of the cerebral cortex. These images were obtained from a living animal using μODT at a wavelength of 1300 nm.^8^ This demonstrates the high sensitivity of OCT techniques in visualizing cerebrovascular structures and providing quantitative neurovascular CBF imaging *in vivo*.

### 2.3 High Spatial Resolution to Permit Separation of Vascular Components (e.g., Artery, Vein, and Capillary) and Image the Vascular Hemodynamic Changes

The ability to distinguish between veins and arteries is crucial for understanding their individual responses to various physiological, pharmacological, and functional stimuli. Such differentiation is possible with 3D μODT.^60^ This imaging modality offers high spatial resolution CBF velocity images, detecting vascular networks ranging from large vessels down to microvasculature such as arterioles or capillaries. Figure 2(a) highlights the differentiation of arteries (red-dashed lines) and veins (blue-dashed lines) using μOCA, whereas Fig. 2(b) depicts its 3D ODT of the CBF network image of the rodent cerebral cortex. In addition, MWI offers an alternative for distinguishing arteries (red traces) from veins (blue traces), as seen in Figs. 2(c)–2(d).^59^

### 2.4 High Temporal Resolution to Allow for the Dynamic Assessment of Neuronal and Vascular Responses After an Intervention (e.g., Cocaine Administration)

**Fig. 3:**
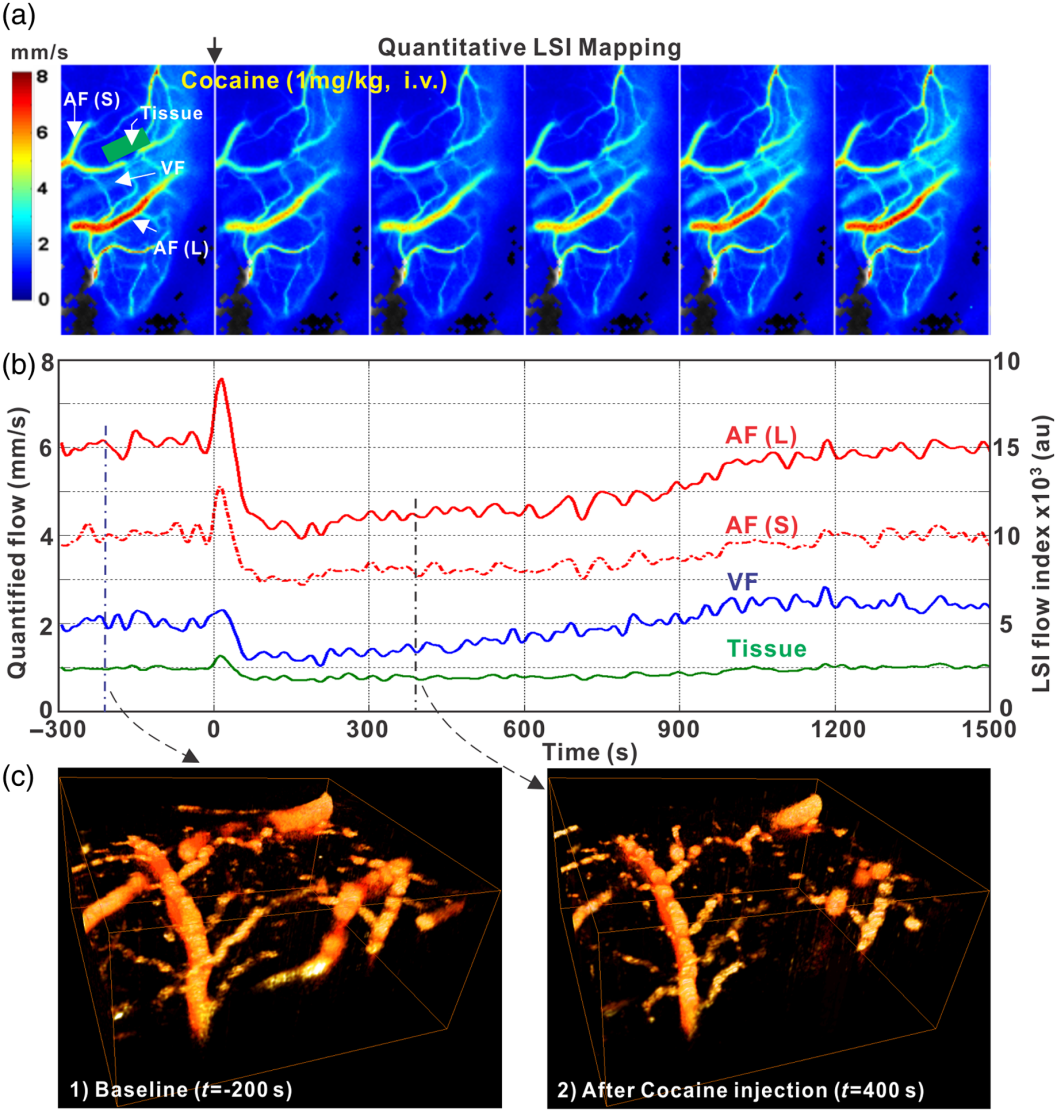
Spatiotemporal dynamics of CBF in the rat cortex in response to cocaine i.v. injection under isoflurane anesthesia using laser speckle contrast imaging (LSCI) and Doppler optical coherence tomography (DRF-OCT). (a) Time course of quantitative flow mapping obtained from LSCI. (b) Cocaine-evoked CBF change from selected larger and small arteriole flows, AF(L) and AF(S), a venule (VF), and an avascular flow or tissue perfusion (tissue) in the ROI by simultaneous imaging of LSCI and DRF-OCT. (c) A pair of 3D DRF-OCT to illustrate vascular constriction after cocaine injection.

Figure 3 presents simultaneous CBF images obtained using LSCI and 3D ODT before and after cocaine administration (1  mg/kg, i.v., a clinically relevant dose). In Fig. 3(a), LSCI captures en face CBF velocity across a large FOV of $5\times 3\;{\mathrm{mm}}^{2}$ at a high frame rate, ∼10 frames per second. This high temporal resolution allows the accurate detection of time-dependent changes in CBF velocity within the entire FOV, which includes both large and small vessels [e.g., labeled AF(L), AF(S)], veins (e.g., labeled VF), and the surrounding cortical tissue (e.g., labeled tissue) resulting from microcirculatory flows irresolvable by LSCI, i.e., <30  μm).^43^ Although LSCI only provides relative flow measurements, its advantage lies in its ability to effectively differentiate CBF indices among vessels of varying calibers.

In parallel, ODT allows for quantitative 3D imaging of the local CBF network with a spatial resolution of less than 10  μm. Figure 3(c) displays a 3D ODT image of the vascular CBF network at baseline (i.e., 200 s before cocaine injection) and 400 s after cocaine injection, indicating that CBF decreased along with vasoconstriction in the cerebrovascular network. These findings demonstrate the effectiveness of integrating LSCI and ODT modalities in detecting changes in CBF induced by cocaine administration as evidenced by the flow rates (represented in pseudo-color) of various vessels regardless of size within the cerebral cortex of a living animal.^43^

### 2.5 Imaging Analysis and Data Quantification

#### 2.5.1 Computation of ${\mathrm{HbO}}_{2}$, HbR, and HbT from Multiwavelength Spectroscopic Imaging (MWI)

Changes in oxygenated (${\mathrm{HbO}}_{2}$) and deoxygenated (HbR) hemoglobin levels can be derived as a function of time based on spectral images simultaneously collected at 568 and 630 nm wavelengths from MWI. The images collected at 568 nm reflect equivalent absorption from both ${\mathrm{HbO}}_{2}$ and HbR in arteries and veins, respectively, whereas those obtained at 630 nm dominantly show HbR in veins. Based on the assumption that the hemodynamics dominate changes in the corresponding light absorption, $\Delta [{\mathrm{HbO}}_{2}]$ and Δ[HbR] are quantified based on the following equations:^52^^,^^53^^,^^61^

$$[\begin{array}{c} \Delta [{\mathrm{HbO}}_{2}(t)]\\ \Delta [\mathrm{HbR}(t)] \end{array}]=[\begin{array}{cc} \varepsilon_{{\mathrm{HbO}}_{2}}^{\lambda_{1}} & \varepsilon_{\mathrm{HbR}}^{\lambda_{1}}\\ \varepsilon_{{\mathrm{HbO}}_{2}}^{\lambda_{2}} & \varepsilon_{\mathrm{HbR}}^{\lambda_{2}} \end{array}{]}^{-1}\times [\begin{array}{c} \frac{\ln(R_{\lambda_{1}}(0)/R_{\lambda_{1}}(t))}{L_{\lambda_{1}}(t)}\\ \frac{\ln(R_{\lambda_{2}}(0)/R_{\lambda_{2}}(t))}{L_{\lambda_{2}}(t)} \end{array}].$$

where ε indicates the molar spectral absorbance coefficient at the wavelengths, $\lambda_{1}=568\;\mathrm{nm}$ and $\lambda_{2}=630\;\mathrm{nm}$, and $R_{\lambda_{1}}$ and $R_{\lambda_{2}}$ refer to the measured diffuse reflectance matrices at time t, to be divided by their respective path lengths, $L_{\lambda_{1}}$ and $L_{\lambda_{2}}$. Accordingly, total hemoglobin changes [ΔHbT] in veins, arteries, and tissue with microvasculature within the cortex area can also be calculated from the sum of $\Delta [{\mathrm{HbO}}_{2}]$ and Δ[HbR] to examine total blood volume changes in specific types of vessels at high resolutions, as demonstrated in Figs. 1 and 5(i).

#### 2.5.2 Neuronal/astrocytic $[{\mathrm{Ca}}^{2+}{]}_{i}$ fluorescence changes from MWI

Intracellular $[{\mathrm{Ca}}^{2+}{]}_{i}$ fluorescence intensity changes (ΔF(t)/F) from neuronal or astrocytic GECIs can be quantified based on their reflectance intensity relative to baseline (e.g., before cocaine), $[F(t)-F_{\text{baseline}}]/F_{\text{baseline}})\times 100$ (%),^47^ as illustrated in Figs. 6(d), 6(h), 7(b), 7(g), and 8(c). In addition, these ${\mathrm{Ca}}^{2+}$ signal changes can be visually represented through ratio images, as shown in Figs. 6(c), 6(g), 7(c), 7(d), and 7(f), to demonstrate spatial changes of ${{\mathrm{Ca}}^{2+}}_{N}$ and/or ${{\mathrm{Ca}}^{2+}}_{A}$ in a large FOV.

#### 2.5.3 ΔCBF from laser speckle contrast imaging

The integration of LSCI into MWI enables quick detection of the relative CBF (ΔCBF) changes using spectral images collected at 830 nm by acquiring signals on moving scatterers such as red blood cells within vessels. The LSCI images are processed by spatial or/and temporal pixel binning to enhance SNR,^48^ and the following speckle contrast constant and formula are used to quantify relative ΔCBF:^48^^,^^55^^,^^62^ $K=\frac{\sigma }{\left\langle I\right\rangle }$ where

$$K={\left\{\frac{\tau_{c}}{T}+\frac{\tau_{c}^{2}}{2T^{2}}[\exp(-\frac{2T}{\tau_{c}})-1]\right\}}^{\frac{1}{2}},$$

$$\tau_{c}={\left[\frac{2\pi \alpha }{\lambda }\cdot {\left\langle v^{2}\right\rangle }^{\frac{1}{2}}\right]}^{-1}.$$

where ⟨I⟩ and σ refer to the mean and standard deviation of the intensities within a selected sub-volume. T indicates the camera exposure time, and $\tau_{c}$ is the decorrelation time of LSCI. Accordingly, the LSCI recording offers mapping of time-dependent ΔCBF at high temporal resolution, as seen in Figs. 3(a) and 3(b).

#### 2.5.4 Image reconstruction and quantitative CBF velocity from OCT

**Fig. 4:**
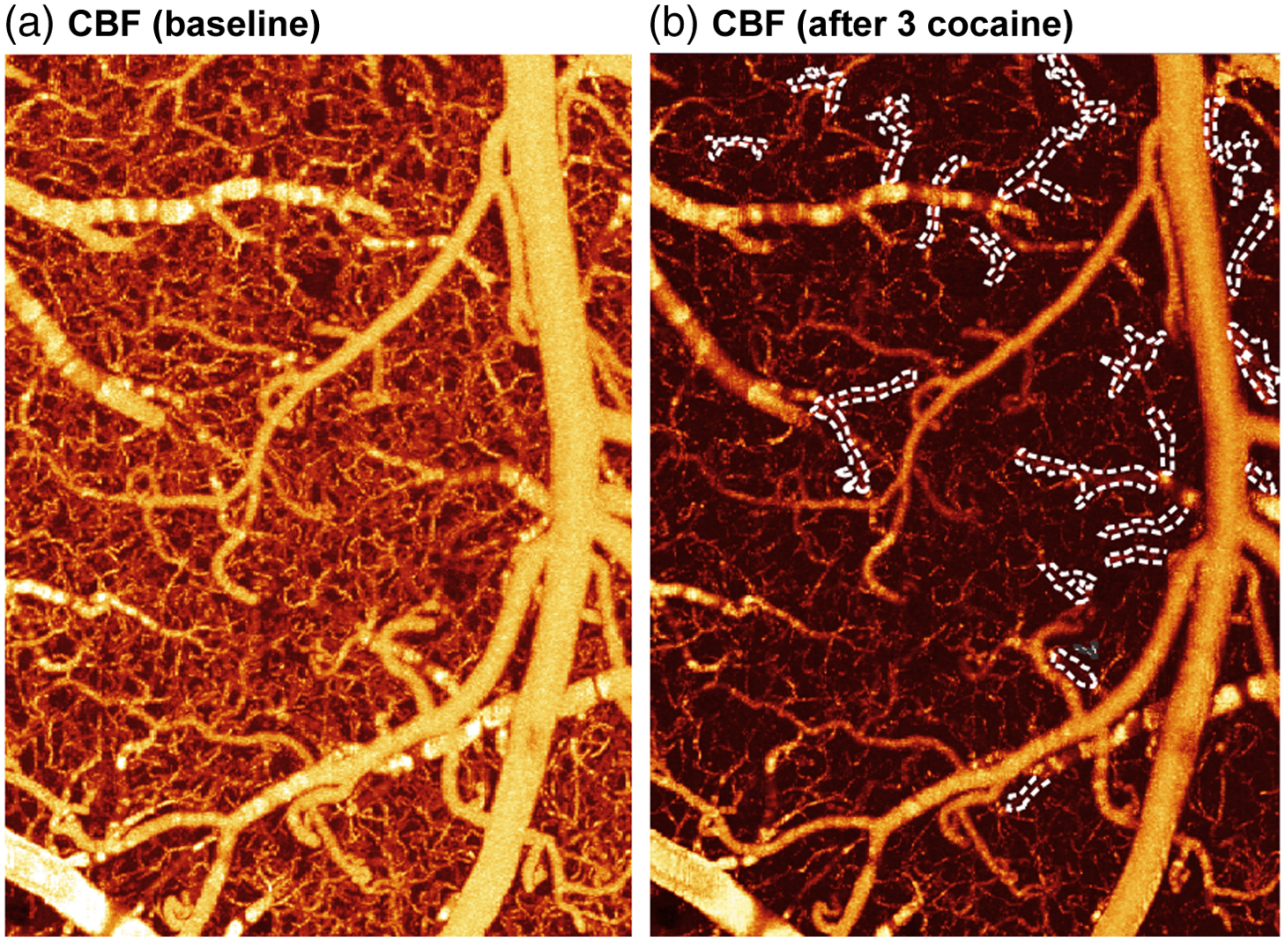
Observation of cocaine-induced micro-ischemia from a mouse cortex. (a) μODT image shows the cortical blood flow at baseline. (b) μODT image after three sequential cocaine administrations, white-dashed traces show the occluded vessels after three cocaine administrations.

**Fig. 5:**
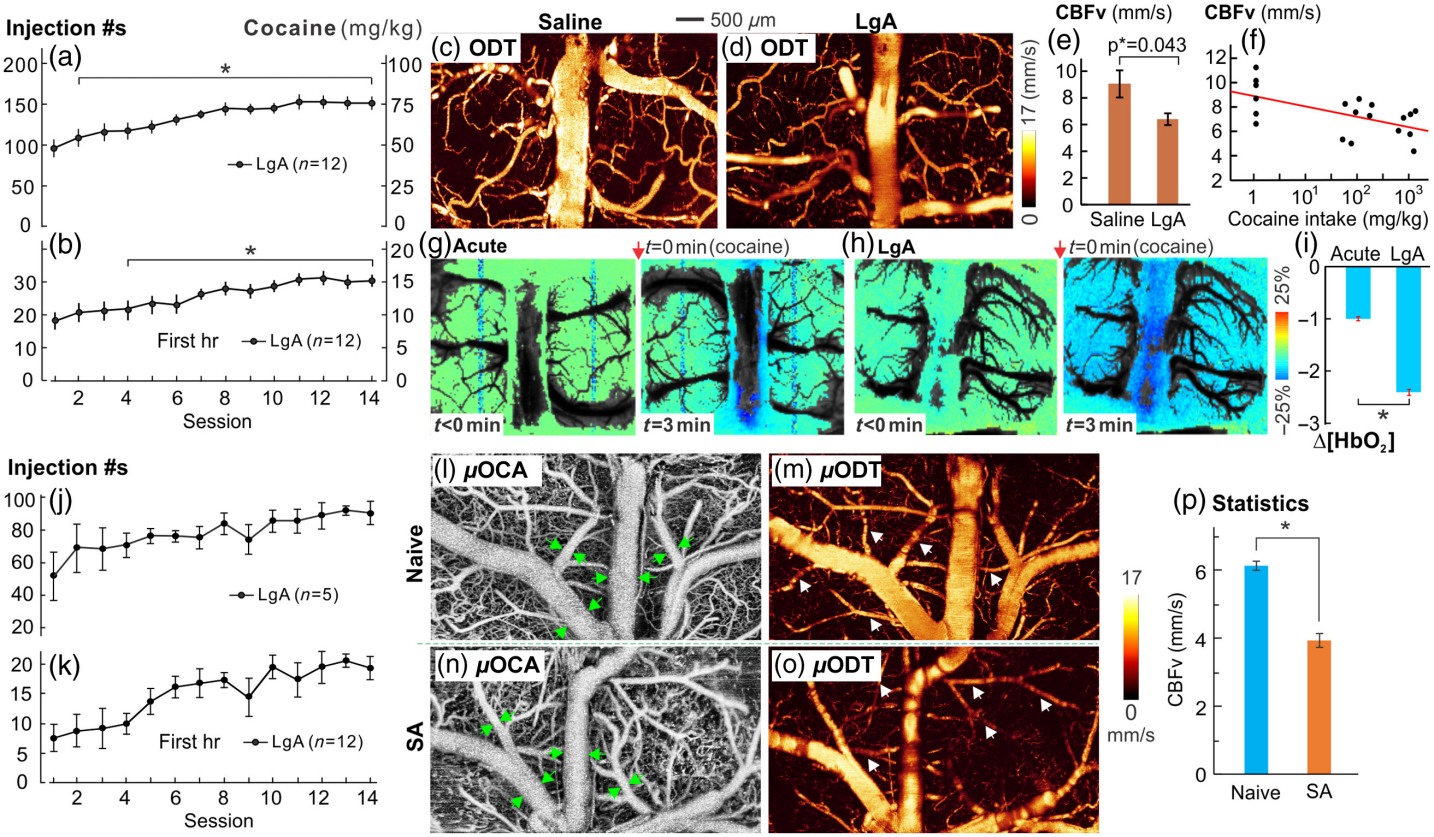
Cocaine self-administration (SA) by rats (a), (b) and by mice (j), (k), indicating the escalation period of cocaine intake. LgA: cocaine infusions in 6 h session. (c)–(i) Image comparison between the rat control group with saline and cocaine SA rats for assessing the effects of LgA cocaine SA on the prefrontal cortex (PFC). (c), (d) Representative images of the rat control and LgA groups showing their quantitative CBF velocity in the PFC, respectively. The LgA animal exhibited a global CBF velocity decrease. (e) Significant CBF velocity decreases in vessels between control (n=6) and LgA (n=6) rats. (f) Correlation between CBF velocity and doses of cocaine intake (in log10 scale), indicating an inverse association between CBF velocity and the amount of cocaine administered by the animals. (g) Representative full-field $\left[{\mathrm{HbO}}_{2}\right]$ map of the PFC before and 3 min after acute cocaine (1  mg/kg, i.v.) in a cocaine-naïve (i.e., control) and in a LgA rat in panel (h), indicating a global decrease in $\Delta [{\mathrm{HbO}}_{2}]$ of the LgA cocaine animal (represented by the blue area in the PFC image at t=3  min). (i) Comparison of the total decrease of $\left[{\mathrm{HbO}}_{2}\right]$ over the recording period (t=30  min) in response to acute cocaine (1  mg/kg, i.v., during imaging) between the control and LgA rats, showing significant $\left[{\mathrm{HbO}}_{2}\right]$ decreases in LgA cocaine animal brain (p=0.02). (Updated from Du et al.^59^). (j)–(p) Mouse model of LgA SA (n=5) to compare with its controls (saline infusion, n=5). (l), (n) 3D μOCA for microvasculature in control and SA mice, indicating cocaine-induced vasoconstriction in the PFC of SA mouse (green arrows); (m), (o) μODT for quantitative CBF in control and SA mice, indicating a global CBF decrease in the PFC of SA mouse. (p) Statistical comparison of CBF in vessels (white arrows between controls (n=5) and SA (n=5) mice.

Quantitative 3D CBF velocity network maps from μODT shown in Figs. 2(b), 3(c), 4, 5(c), 5(d), 5(m), and 5(o) are generated using the phase subtraction method (PSM), which calculates an apparent Doppler CBF velocity (v) based on a phase shift occurred by moving scatters (i.e., red blood cells) between two consecutive A-scans of OCT^63^^,^^64^

$$v=\frac{\lambda_{0}\Delta \phi_{\max}}{4{\pi \mathrm{nT}\;\cos\;\theta }_{z}}.$$

Here, $\lambda_{0}$ is the central wavelength, and n is the refractive index of the brain tissue (1.38). T refers to the time interval between two adjacent A-scans, and $\theta_{z}$ is the flow angle with respect to the incident light. Based on this calculation, apparent CBF velocity can be examined in veins, arteries, and even capillaries in 3D.

## 3 Imaging Cocaine’s Effects on Neurovascular Network and Tissue Oxygenation

### 3.1 Repeated Cocaine Induces Microischemia

The ability to monitor dynamic CBF networks is crucial for tracking temporal variations in the neurovascular system, which are often heterogeneous and fluctuating over time. The ultrahigh-resolution μOCA/μODT^60^^,^^63^ has allowed us to explore cocaine’s effects on small vessels such as capillaries within the mouse brain. In addition, with its rapid acquisition capability and a real-time construction of an ongoing ODT scan, abnormal changes in CBF such as localized ischemia within the brain can be immediately detected.^64^ Figure 4 illustrates the effects of cocaine on the neurovascular network, showing a significant reduction in CBF, particularly within terminal micro-vessels, which led to local ischemia. Specifically, Fig. 4(a) is an μODT image showing the cortical blood flow before cocaine, and Fig. 4(b) illustrates the vascular response to three sequential cocaine injections (2.5  mg/kg/each, *i.v*., ∼45  min apart from each other), which documented the disruption of CBF in downstream micro-vessels and microischemia due to cocaine-induced vasoconstriction in the cortex. These microischemic events, being highly localized, often go undetected by current imaging techniques including μOCA, which showed no evident disruptions even after administering three consecutive doses of cocaine.^63^ Therefore, this approach provides a systematic way to evaluate the impact of both acute and repeated cocaine administration on vascular architecture and CBF. Understanding these changes is crucial for elucidating the mechanisms underlying cocaine-induced ischemic and hemorrhagic strokes, thus providing an imaging tool to monitor potential therapeutic interventions.

### 3.2 Chronic Cocaine Decreases CBF Across Vascular Trees and Induces Brain Hypoxia

Studies of addiction-like behaviors commonly use rodent models involving self-administration (SA) of cocaine as it leads to high doses of cocaine intake in rats, thereby providing insights into human cocaine addiction.^65^ In addition, addiction-related behaviors including tolerance, increased drug intake, and heightened motivation are replicated in rodent models that have unrestricted access to cocaine.^66^^,^^67^ Especially, compulsive drug consumption is recapitulated in extended access, such as long-access (LgA, e.g., a daily 6-h session) SA.^68^ We used this compulsive intravenous cocaine SA model in rats.^7^ Rats were allowed to self-administer i.v. cocaine with extended access (0.5  mg/kg/ injection, 6  h/day, LgA). This LgA increased the intake and motivation to seek and take cocaine [Fig. 5(a)], as reflected in the increased injection #s even for the first hour of each session [Fig. 5(b)]. We used quantitative imaging of CBF to assess neurovascular networks within the PFC of LgA animals, compared with those of yoked controls that received saline infusion instead of cocaine. Figures 5(c) and 3(d) illustrate the CBF velocity measurements, revealing significantly reduced flow throughout the cerebrovascular network in the PFC of LgA animals compared with that of the controls. Statistical comparisons show a significant difference between these two groups [Fig. 5(e)]. Correlation analyses [in Fig. 5(f)] show a significant inverse regression (r=−0.6, p<0.01), indicating that rats that self-administered larger cocaine doses had greater CBF reductions.

Figures 5(g)–5(h) show the spatiotemporal changes in oxygenated-hemoglobin ($\Delta [{\mathrm{HbO}}_{2}]$) in the PFC following an acute cocaine challenge (1  mg/kg, i.v.) and compare baseline images to those taken at 3 min post-cocaine injection in both control and LgA animals. In control (drug-naïve) animals, acute cocaine administration resulted in a decrease in $\Delta [{\mathrm{HbO}}_{2}]$ within 3 min, primarily localized around the central vein in the PFC [Fig. 5(g)]. By contrast, the reduction in $\Delta [{\mathrm{HbO}}_{2}]$ was more extensive in LgA animals, affecting the entire FOV of the PFC [Fig. 5(h)]. The statistical comparison showed that the decrease in $\Delta [{\mathrm{HbO}}_{2}]$ in LgA animals was twice as large as that observed in controls [Fig. 5(i)], suggesting that chronic cocaine exposure led to significant hypoxia in the PFC.

Recently, we extended this cocaine SA model to mice. LgA mice were allowed to self-administer cocaine for 6 h/day, and this LgA access likewise increased their motivation to take more cocaine [Figs. 5(j)–5(k)]. Figures 5(i)–5(n) compare their 3D microvasculature while Figs. 5(m) and 5(o) compare their CBF in the PFC with vasoconstriction [Fig. 5(n)] and drastic CBF decreases [Fig. 5(o)] in cocaine SA compared with naïve mice. Statistical analysis indicated that CBF velocity (3.92±0.20  mm/s) in SA mice was significantly lower than that in naïve mice (6.12±0.13  mm/s; p*=0.012; ROIs: m=5/animal, n=5), documenting that cocaine reduced CBF in the PFC. This result agrees with the observation from the rat models above and also is akin to what has been reported in humans.

Chronic cocaine misuse in humans has consistently been linked to reduced CBF and diminished cortical perfusion.^5^^,^^69^ Evidence from both PET and single-photon emission computed tomography (SPECT) studies revealed that individuals with chronic cocaine misuse exhibit perfusion deficits comparable with those observed in stroke or head injury patients.^70^ These abnormalities in CBF are further associated with changes in neuropsychological function and the presence of neurologic symptoms in cocaine users^71^^–^^73^ including cocaine-induced strokes, which can occur even in young individuals.^74^

## 4 Imaging Cocaine’s Effects on Neuronal Activities

As mentioned above, brain imaging revealed a marked decrease in CBF in individuals with cocaine use compared with healthy, non-drug users.^5^ These deficits in CBF are believed to play a major role in the cognitive decline observed in chronic cocaine users and are consistent with reports of young patients experiencing neurological issues due to cocaine use.^75^^,^^76^ The application of advanced optical imaging technologies has provided detailed insights into the effects of cocaine on both cerebral vasculature and neuronal activity in animal models, allowing for a more nuanced understanding of its impact on cerebral hemodynamics and neurovascular function.

Investigating cocaine’s impact on neurons requires measuring intracellular free calcium (${{\mathrm{Ca}}^{2+}}_{N}$), as ${{\mathrm{Ca}}^{2+}}_{N}$ transients are critical indicators of neuronal activity.^77^ Recently, *in vivo* ${{\mathrm{Ca}}^{2+}}_{N}$ fluorescence imaging has advanced the understanding of neuronal activity and circuit dynamics underlying the neurobiology of addiction.^35^^,^^37^ In our studies, we integrated multiwavelength spectral images and neuron-specific ${{\mathrm{Ca}}^{2+}}_{i}$ fluorescence imaging to build the MIP, and this multimodal approach (shown in Fig. 1) allows for concurrent detection of hemodynamic changes such as in CBF, CBV/HbT, ${\mathrm{HbO}}_{2}$ alongside ${{\mathrm{Ca}}^{2+}}_{N}$ fluorescence from the cortical brain *in vivo*. This integrated technology makes it possible to differentiate neuronal activity from cerebrovascular changes triggered by cocaine, providing a more comprehensive understanding of the drug’s effects on the brain.

### 4.1 Cortical Neuronal Responses to Cocaine: Awake Versus Anesthetized Animals

**Fig. 6:**
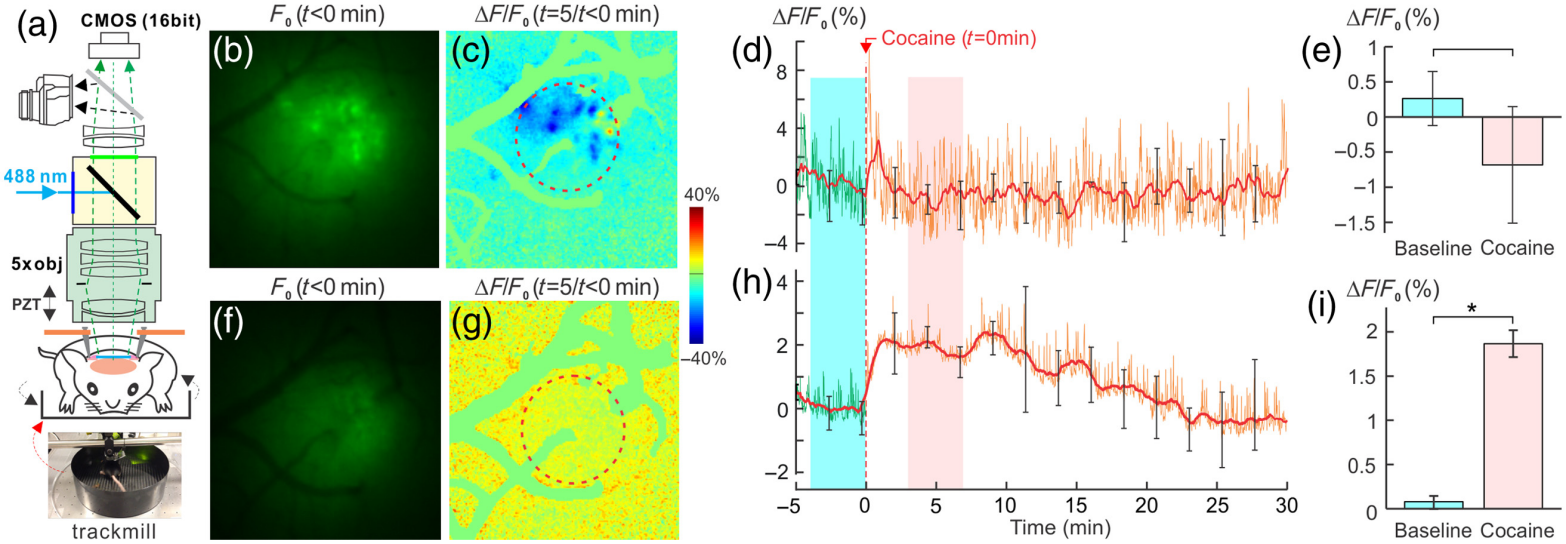
(a) MIP with air-floating mobile cage to image cocaine-induced neuronal ${{\mathrm{Ca}}^{2+}}_{N}$ changes in the awake (b)–(e) and anesthetized states (f)–(i). (b), (f) ${\mathrm{Ca}}^{2+}$ fluorescence images in awake- and isoflurane-anesthetized-states, respectively. (c), (g) The ratio images before and after cocaine in the awake- (c) and isoflurane-anesthetized states (g). (d), (h) Time-lapse $\Delta {{\mathrm{Ca}}^{2+}}_{N}$ (%) in the awake- (d) and anesthetized states (h). (e)–(i) Mean ${{\mathrm{Ca}}^{2+}}_{N}$ differences between baseline (t=−5 to 0 min) and after cocaine (t=3 to 8 min), showing that the mean ${{\mathrm{Ca}}^{2+}}_{N}$ fluorescence was not significantly changed in the awake state [F(14,28)=1.1, p=0.39], whereas individual neuronal activity was decreased after cocaine (c). Mean ${{\mathrm{Ca}}^{2+}}_{N}$ fluorescence was increased (1.86±0.15%, [F(14,56)=8.16, p<0.001, n=5] in the anesthetized state (updated from Park et al.^78^).

Most preclinical imaging studies to explore cocaine’s effects on the brain have been conducted under anesthesia with the advantage of minimizing motion artifacts, but they can potentially introduce confounding variables. To address this issue, we used optical imaging to compare cocaine’s effects in awake versus isoflurane-anesthetized mice. We customized an air-floating mobile cage (a mouse track mill) to be compatible with the MIP, as shown in Fig. 6(a). For detecting neuronal ${{\mathrm{Ca}}^{2+}}_{N}$ fluorescence, we used genetically encoded ${{\mathrm{Ca}}^{2+}}_{i}$ indicator, GCaMP6f (AAV1-Syn-GCaMP6f-WPRE-SV40, provided by PENN Vector Core), which was virally delivered into the somatosensory cortex (SSC) of each mouse. Approximately ∼4 weeks following the GCaMP6f delivery, we implanted a multilayer cranial window over the SSC to enable optical imaging.^78^^,^^79^

Figure 6(a) illustrates MIP with an air-floating mobile cage for awake and anesthetized mice. Figures 6(b) and 6(f) present ${{\mathrm{Ca}}^{2+}}_{N}$ fluorescence images from the SSC of a mouse in awake- and isoflurane-anesthetized states (∼2.5%), respectively. Figure 6(c) represents the ratio image comparing the post-cocaine fluorescence (t=3 to 8 min) to the baseline (t=−5 to 0 min) in an awake animal, whereas Fig. 6(g) shows the ratio image from an anesthetized animal. The time course ${{\mathrm{Ca}}^{2+}}_{N}$ in terms of fluorescence intensity change ($\Delta F(t)/F_{0}\%$) in the selected regions of interest (ROIs), marked by dashed circles in Figs. 6(c) and 6(g), is displayed in Figs. 6(d) and 6(h) for both awake and anesthetized states, including the mean ${{\mathrm{Ca}}^{2+}}_{N}$ fluorescence (solid lines) and its fluctuations (spikes) over time. Figures 6(e) and 6(i) present the statistical results of the mean ${{\mathrm{Ca}}^{2+}}_{N}$ changes averaged over 5 min before and after cocaine [highlighted by dashed boxes in Figs. 6(d) and 6(h), respectively]. It showed a nonsignificant decrease from its baseline in the awake animals [0.26±0.38% at baseline versus −0.72±1.32% after cocaine, p=0.39]; n=3; Fig. 6(e)]. By contrast, in the anesthetized state, the mean ${{\mathrm{Ca}}^{2+}}_{N}$ fluorescence significantly increased [from a baseline of 0.07±0.07% to 1.86±0.15% after cocaine administration; p<0.001; n=5; Fig. 6(i)]. These findings suggest that neuronal responses to cocaine are significantly influenced by isoflurane anesthesia, emphasizing the need to use awake animal models or other anesthetics that have a smaller impact on neuronal and vascular function (e.g., dexmedetomidine, α-chloralose^50^^,^^51^^,^^55^^,^^56^) for studying the effects of cocaine in the brain.

## 5 Imaging Cocaine’s Effects on Brain Connectivity Between the Ventral Tegmental Area and Prefrontal Cortex

The PFC is influenced by dopaminergic and glutamatergic neurons originating from the VTA and disruption of this connection can lead to impulsive behaviors, such as those seen during cocaine intoxication. To investigate cocaine’s effects on connectivity between PFC and VTA, we studied the reactivity of the PFC to direct VTA stimulation following acute cocaine (30  mg/kg, i.p.).^80^ Using a genetically encoded calcium indicator (GCaMP6f), we tracked neural activity in mPFC in response to “tonic-like” (5 Hz) and “phasic-like” (50 Hz) electrical stimulation of the VTA. The 5 and 50 Hz stimuli were used to mimic DA neuronal tonic and phasic neuronal firing in the VTA, respectively.^81^ Tonic firing (1 to 5 Hz) maintains low, stable levels of extracellular DA, which are crucial for sustained processing and motivation. By contrast, phasic firing (40 to 50 Hz) occurs in bursts and induces a sharp increase in DA concentration, which is important for reward, conditioning,^82^^–^^84^ and response to drugs, such as cocaine.^85^

### 5.1 PFC Responses to VTA Tonic-Like Stimulation with or Without Cocaine

**Fig. 7:**
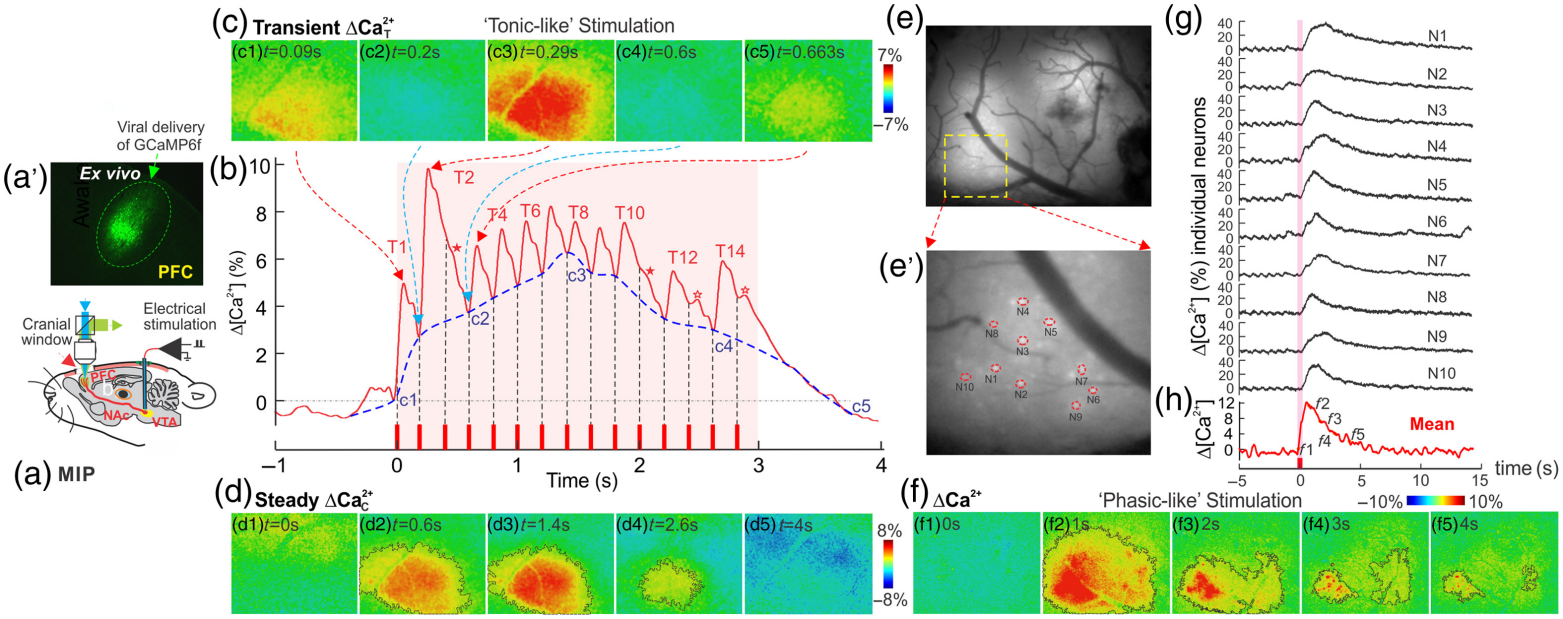
Dynamic recording of neuronal ${\mathrm{Ca}}^{2+}$ in the medial prefrontal cortex (mPFC) in response to tonic-like (0.3  mA/5  Hz/3  s, 15 pulses) and phasic-like (0.3  mA/50  Hz/0.3  s, 15 pulses) ventral tegmental area (VTA) stimulation. (a) The experimental setup showing MIP. (a’) GCaMP expression localized in mPFC. (b) Fluorescence change $\Delta {\mathrm{Ca}}^{2+}$ (solid red trace) evoked by a tonic-like stimulation (the blue-dashed trace indicates the accumulative increase $\Delta {{\mathrm{Ca}}^{2+}}_{C}$). (c) Timelapse images of transient responses ${{\mathrm{Ca}}^{2+}}_{T}$ to individual pulse series of ‘tonic-like’ stimulation. (d) Spatial-temporal evolution of neuronal calcium $\Delta {{\mathrm{Ca}}^{2+}}_{C}$ reactivity. (e)–(g) Phasic-like VTA stimulation. (e) A representative image of neuronal ${\mathrm{Ca}}^{2+}$ in mPFC during VTA stimulation (t≈0.2  s). (f1–f5) Timelapse images of accumulative ${\mathrm{Ca}}^{2+}$ increase before (t<0  s) and after phasic-like VTA stimulation (t>0  s). (h) The temporal profile of $\Delta {\mathrm{Ca}}^{2+}(t)$ during a phasic-like stimulation. (e’) A zoomed-in version of the yellow-dashed box in panel (e), demonstrating cellular resolution to capture individual GCaMP6f neuronal ${\mathrm{Ca}}^{2+}$ signals (n=1,…,10). (g) Intracellular $\Delta {\mathrm{Ca}}^{2+}(t)$ within these 10 neurons (N=1,…,10) during a phasic-like stimulation. They showed a highly synchronized, strong response (modified from Park et al.^80^).

Figure 7 shows representative neuronal ${{\mathrm{Ca}}^{2+}}_{N}$ fluorescence transients in the mPFC evoked by tonic-like and phasic-like VTA stimulation, Specifically, Fig. 7(b) presents the response to the stimulation (0.3  mA/5  Hz/3  s, 15 pulses) from one animal. During the VTA stimulation period (marked by the pink-shaded area), a gradual, accumulative increase in calcium fluorescence intensity above baseline (${{\mathrm{Ca}}^{2+}}_{C}$, represented by the blue-dashed trace) was observed, and it reached a peak of ΔF/F>6% at c3 (t=1.4  s). The spatiotemporal evolution of neuronal reactivity in the mPFC is illustrated in Fig. 7(c), indicating that the PFC neuronal response was closely synchronized with the applied stimuli to VTA. In Fig. 7(d), the accumulation of neuronal ${{\mathrm{Ca}}^{2+}}_{N}$ in the mPFC during tonic-like VTA stimulation is depicted. Alongside the strong transient response (${{\mathrm{Ca}}^{2+}}_{T}$) to each pulse, there was also a rapid increase in baseline ${\mathrm{Ca}}^{2+}$ (i.e., ${{\mathrm{Ca}}^{2+}}_{C}$), which peaked at time point c3 (t=1.4  s) before gradually declining after the VTA stimulation ended. Following cocaine administration, however, the sensitivity of the mPFC to tonic-like VTA stimulation was notably reduced, with a 50% reduction in the number of ${{\mathrm{Ca}}^{2+}}_{T}$ transients in response to individual electrical stimuli. In addition, the recovery time of accumulated ${{\mathrm{Ca}}^{2+}}_{C}$ influx (${{\mathrm{Ca}}^{2+}}_{C}$) was prolonged.^80^

### 5.2 PFC Responses to VTA Phasic-Like Stimulation with or Without Cocaine

The phasic-like stimulation was conducted using the stimulation paradigm of 0.3  mA/50  Hz/0.3  s, 15 pulses at the VTA. There were no ${{\mathrm{Ca}}^{2+}}_{T}$ transients detected in the mPFC for individual stimuli. Instead, a cumulative ${{\mathrm{Ca}}^{2+}}_{C}$ increase was observed, and it peaked shortly after the stimulation ended, followed by the decay toward the baseline. Figure 7(e) shows a neuronal ${{\mathrm{Ca}}^{2+}}_{C}$ fluorescence image for one animal responding to the phasic-like VTA stimulation period at ∼t=0.2  s, Figs. 7(f1)–7(f5) depict the spatiotemporal evolution of neuronal reactivity in the mPFC, with a steady-state increase in ${{\mathrm{Ca}}^{2+}}_{C}$ fluorescence over the baseline ($\Delta {{\mathrm{Ca}}^{2+}}_{c}$). Interestingly, unlike the spatially uniform rise and decay in ${{\mathrm{Ca}}^{2+}}_{C}$ seen with tonic-like stimulation [as shown in Fig. 7(d)], the ${{\mathrm{Ca}}^{2+}}_{C}$ change evoked by phasic-like stimulation was more localized and patchier. Figure 7(e’) offers a close-up view of the region outlined by the yellow-dashed box in Fig. 7(e). High levels of GCaMP6f expression allowed for the visualization of ${{\mathrm{Ca}}^{2+}}_{c}$ fluorescence in ∼10 distinct neurons (e.g., N1, …, N10), which were randomly selected to track their dynamic responses to the phasic-like VTA stimulation. As plotted in Fig. 7(g), despite minor differences in the response profiles, the $\Delta {{\mathrm{Ca}}^{2+}}_{c}(t)$ changes in these neurons were well synchronized with the VTA stimulation, indicating robust neuronal reactivity in the mPFC to phasic-like stimulation. Cocaine did not alter the peak response, but it did reduce the recovery time to baseline.^80^

These changes in the mPFC play a role in promoting cocaine-binging behavior during intoxication and align with the previous findings of reduced tonic dopamine function in individuals who misuse cocaine.^86^^,^^87^ Such disruptions have been associated with impaired PFC functions, which can contribute to increased drug-seeking behavior and relapse.^88^ Nevertheless, it had been challenging to capture connectivity between the brain regions. Advancements in optical imaging techniques with their high temporal and spatial resolutions have significantly enhanced our understanding of neural circuits.^89^ This includes the ability to capture single neuronal ${\mathrm{Ca}}^{2+}$ transients in response to tonic-like stimulation, as well as neuronal activation triggered by phasic-like VTA stimulation.

## 6 Imaging Cocaine’s Effects on Neuro-Astroglial-Microvascular Network

Although cocaine affects both cerebral vasculature and neuronal activity, it also disrupts astrocytic function, which plays a crucial role in modulating neurovascular coupling—a process that governs cerebral hemodynamics in response to neuronal activation. Clinical studies have identified pathological changes in cocaine users affecting neurons, astrocytes, and vascular structures. These changes include neuronal loss, a reduction in glial fibrillary acidic protein (GFAP)-positive astrocytes, and reactive as well as degenerative changes in the cerebral microvasculature.^90^ In rodent studies, astrocytes were found to restore synaptic glutamate homeostasis in the nucleus accumbens core (NAcore) following repeated cocaine exposure,^91^ and manipulating astrocyte activity was shown to attenuate relapse after cocaine withdrawal. This highlights cocaine’s influence on neuro-astroglial-vascular (NAV) interactions and processes.

The effects of cocaine on cerebral blood vessels, neurons, and astrocytes are complicated by their interactions and the evolving changes that occur with chronic cocaine exposure. This complex nature of NAV affected by cocaine necessitates longitudinal, simultaneous multiparameter measurements conducted *in vivo* to accurately separate and assess these individual effects. However, the *in vivo* examination of astrocytic functions had been challenging because removing astrocytes leads to neuronal death and conventionally used electrophysiological methods cannot be used due to slow changes in the membrane potential of astrocytes.^92^ It has been recently discovered that astrocytic ${{\mathrm{Ca}}^{2+}}_{A}$ signaling can be used as an indicator of their activity because astrocyte activation involves increases in intracellular ${\mathrm{Ca}}^{2+}$; electrophysiological and ${\mathrm{Ca}}^{2+}$ imaging studies using rodent brain slices have provided valuable insights into astrocyte-neuron interactions and their role in neuronal network activity.^93^^–^^95^ Moreover, advancements in *in vivo* imaging techniques such as two-photon microscopy (TPM) have made it possible to monitor astrocyte activity and its relationship with CBF in living brains.^39^ Despite these advances, most ${{\mathrm{Ca}}^{2+}}_{A}$ signaling studies have focused on individual astrocytes within a small FOV accessible by TPM, rather than examining larger populations of astrocytes where synchronized ${{\mathrm{Ca}}^{2+}}_{A}$ signaling occurs.^96^^,^^97^ Consequently, the behavior of synchronized ${{\mathrm{Ca}}^{2+}}_{A}$ signaling in large astrocyte populations, such as astrocyte ensembles, remains poorly understood, as does their functional role in NAV interactions.

In our lab’s work, GECIs such as green GCaMP6f have enabled the measurement of ${\mathrm{Ca}}^{2+}$ fluorescence signals from cortical neurons and astrocytes in separate animal groups.^55^ More recently, jRGECO1a, a sensitive red GECI, has been employed for imaging neuronal activity.^50^^,^^98^^,^^99^ Building on this technique, an approach of combining red jRGECO1a with green GCaMP6f has been used to simultaneously capture both neuronal and astrocytic activities within the same animal and to explore their interactions. Consequently, we developed a fluorescence imaging system capable of distinguishing these two distinct fluorescence emissions spectrally and integrated it with ultra-high-resolution optical coherence Doppler tomography to establish a multichannel fluorescence and μOCA/μODT microscope (*fl-ODM*). This multimodal system enabled us to simultaneously image large-scale ${\mathrm{Ca}}^{2+}$ fluorescence in astrocytes and neurons, as well as visualize 3D CBF velocity within vascular networks of the mouse PFC.

**Fig. 8:**
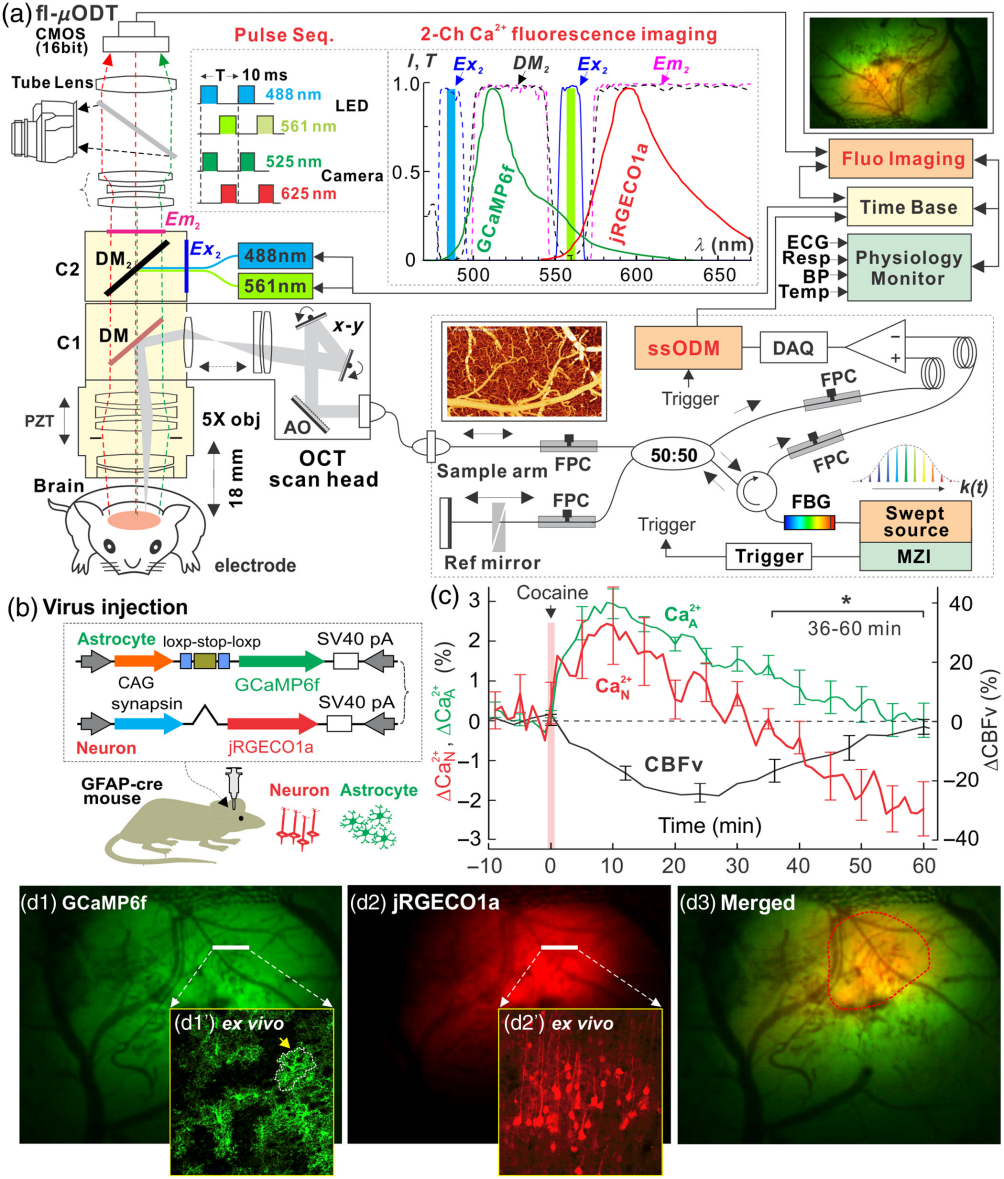
(a) fl-ODM imaging system integrated 3D μOCA/μODT and two-channel fluorescence imaging for simultaneous imaging microvasculature and CBF velocity within FOV of $2.4\times 2\times 1.2\;{\mathrm{mm}}^{3}$ and neuronal and astrocytic ${\mathrm{Ca}}^{2+}$ fluorescence imaging ($4\times 3\;{\mathrm{mm}}^{2}$) of the mouse cortex. It was modified based on a Nikon FN-1 microscope using a broadband 5× obj. (e.g., LSM03, Thorlabs). (b) Viral injection to express ${\mathrm{Ca}}^{2+}$ in astrocytes (${{\mathrm{Ca}}^{2+}}_{A}$, GCaMP6f) and neurons (${{\mathrm{Ca}}^{2+}}_{N}$, jRGECO1a) in the cortex of GFAP-cre mice). (c) Mean $\Delta {{\mathrm{Ca}}^{2+}}_{A}$ (green), $\Delta {{\mathrm{Ca}}^{2+}}_{N}$ (red), and vascular ΔCBF velocity (black) responses to cocaine (1  mg/kg, i.v.), to compare cocaine’s effects on neuronal ${{\mathrm{Ca}}^{2+}}_{N}$, astrocytic ${{\mathrm{Ca}}^{2+}}_{A}$ fluorescence, and vascular CBF velocity in the PFC (n=7 mice). (d1–d3) *In vivo* images of ${{\mathrm{Ca}}^{2+}}_{A}$ (GCaMP6f), ${{\mathrm{Ca}}^{2+}}_{N}$ (jRGECO1a) channels, and the merged images with the *ex vivo* evidence of cell-specific expressions (d1’, d2’) (updated from Du et al.^47^).

Figure 8(a) illustrates the design of a custom-built fl-ODM system,^47^ which integrates two imaging modalities into a single upright microscope (FN1, Nikon) through epi-fluorescence cube turrets $\left(C_{1},C_{2}\right)$ for *in vivo* small animal studies. The μOCA and μODT systems in the NIR range (λ=1.3  μm, Δλ=230  nm) were incorporated via a dichroic mirror ($\lambda_{1\mathrm{DM}}=1.1\;\mu m$) within $C_{1}$. This system provides three-dimensional images of the microvasculature structures and quantitative measurements of CBF velocity across vascular networks in the mouse cortex over a large FOV ($∼2.4\times 2\times 1.2\;{\mathrm{mm}}^{3}$) with capillary-level resolution (<5  μm). The technical specifications of 3D μOCA/μODT were previously reported,^100^ with modifications to include a custom high-fidelity 2D confocal laser scanning module to link the μOCT engine to fl-ODM. The two-channel spectral imaging of the synchronized intracellular calcium fluorescence in astrocytes (${{\mathrm{Ca}}^{2+}}_{A}$ expressed with GCaMP6f: $\lambda_{\mathrm{EX}1}=485\pm 12\;\mathrm{nm}$, $\lambda_{\mathrm{DM}1}=495\;\mathrm{nm}$, $\lambda_{\mathrm{EM}1}=520\pm 20\;\mathrm{nm}$) and neurons (${{\mathrm{Ca}}^{2+}}_{N}$ expressed with jRGECO1a: $\lambda_{\mathrm{EX}2}=559\pm 8\;\mathrm{nm}$, $\lambda_{\mathrm{DM}2}=573\;\mathrm{nm}$, $\lambda_{\mathrm{EM}2}\geq 574\;\mathrm{nm}$) over a broad FOV ($∼4\times 3\;{\mathrm{mm}}^{2}$) is achieved through a custom epifluorescence cube ($C_{2}$). The system uses pulsed high-power narrow-band blue (488 nm) and yellow-green (560 nm) LEDs from a light engine (Aura III, Lumencor) for time-sharing spectral excitation, synchronized with a SCMOS camera (Zyla 5.5, Andor) to acquire sequential fluorescence images, with an exposure time of 10-ms exposure per channel. Neuronal ${{\mathrm{Ca}}^{2+}}_{N}(t)$ and astrocytic ${{\mathrm{Ca}}^{2+}}_{A}(t)$ activities, recorded at up to 80 frames per second, were calculated as the relative fluorescence changes (ΔF/F) compared with baseline levels to represent respective activities.

Figure 8(b) depicts our method for expressing astrocytic ${{\mathrm{Ca}}^{2+}}_{A}$ and neuronal ${{\mathrm{Ca}}^{2+}}_{N}$ in the PFC *in vivo*. We used a GFAP-Cre mouse model combined with the delivery of two mixed viral vectors, 50% AAV5.CAG.Flex.GCaMP6f.WPRE.SV40 (#100835, Add-gene) and 50% AAV1.Syn.NES-jRGECO1a.WPRE.SV40 (100854, Add-gene). This combination enabled the expression of GCaMP6f for ${{\mathrm{Ca}}^{2+}}_{A}$ fluorescence in astrocytes and jRGECO1a for ${{\mathrm{Ca}}^{2+}}_{N}$ fluorescence in neurons within the cortex. Before imaging, a cranial window was surgically implanted over the PFC.^79^ Figures 8(d1)–8(d3) present simultaneously captured *in vivo* images of ${{\mathrm{Ca}}^{2+}}_{A}$ [Fig. 8(d1) and ${{\mathrm{Ca}}^{2+}}_{N}$ [Fig. 8(d2)] fluorescence in the PFC of a GFAP-cre mouse at   4 weeks after viral injection. *Ex vivo* double staining using GFAP antibodies to label astrocytes and NeuN antibodies to label neurons validated the specificity of ${\mathrm{Ca}}^{2+}$ expression in astrocytes [Fig. 8(d1’)] and neurons [Fig. 8(d2’)].

Figure 8(c) presents simultaneous measurements of changes in neuronal calcium (${{\mathrm{Ca}}^{2+}}_{N}$), astrocytic calcium (${{\mathrm{Ca}}^{2+}}_{A}$), and CBF velocity within the PFC induced by cocaine, as observed in seven animals. It shows that cocaine led to an increase in $\Delta {{\mathrm{Ca}}^{2+}}_{N}$ (2.46±0.88%), with a peak at 8.2±2.1  min followed by recovery to baseline at 28.8±3.5  min. Similarly, $\Delta {{\mathrm{Ca}}^{2+}}_{A}$ increased to 2.97±0.43% and peaked at 12.1±2.2  min, but it did not return to baseline until 59.5±8.0  min. Concurrently, cocaine reduced mean ΔCBF velocity by −25.1±4.9%, reaching its minimum at 21.0±2.9  min and recovering slowly to baseline at 64.0±7.5  min, which mirrored the duration observed for ${{\mathrm{Ca}}^{2+}}_{A}$. Furthermore, statistical comparisons of the response duration of $\Delta {{\mathrm{Ca}}^{2+}}_{N}$, $\Delta {{\mathrm{Ca}}^{2+}}_{A}$, and ΔCBF velocity to cocaine revealed that the response durations of ${{\mathrm{Ca}}^{2+}}_{A}$ and CBF velocity were significantly longer—approximately twice as long—compared with that of ${{\mathrm{Ca}}^{2+}}_{N}$. No significant difference was found between $\Delta {{\mathrm{Ca}}^{2+}}_{A}$ and ΔCBF velocity response durations (P=0.62). These findings suggest that cocaine increased neuronal ${{\mathrm{Ca}}^{2+}}_{N}$ and astrocytic ${{\mathrm{Ca}}^{2+}}_{A}$ activities while concurrently decreasing CBF velocity in the NAV network. The cocaine-induced changes in CBF velocity were temporally correlated with astrocytic ${{\mathrm{Ca}}^{2+}}_{A}$ activity. Moreover, chemogenetic inhibition of astrocytes during cocaine exposure prevented the vasoconstriction effect and the corresponding decrease in CBF velocity.^46^^,^^47^ In addition, the cocaine-induced increases in neuronal ${{\mathrm{Ca}}^{2+}}_{N}$ were also reduced,^47^ implying that astrocytes play a role in modulating CBF and neuronal responses to cocaine through NAV interaction.

## 7 Other Imaging Modalities to Study Cocaine-Induced Neurovascular Changes in Rodents

Various imaging modalities have provided valuable insights into the dynamic physiology of the brain and its changes induced by stimulants such as cocaine. Although multichannel optical approaches such as MIP and fl-ODM systems developed in our labs demonstrate their significant utility in simultaneously capturing multiple aspects of the neurovascular system, other optical modalities also offer complementary perspectives on cocaine effects on the brain by employing distinct measurement techniques. We searched publications in preclinical research through the database (on PubMed and Google Scholars), focusing on search keywords such as cocaine, addiction, blood flow, vascular, optical imaging, rodents, fiber photometry, two-photon, and GRIN lens, and summarized the details of these studies, such as (1) imaging classification, (2) labeling for vascular/cellular changes, (3) measurements, (4) cocaine doses, (5) effects of cocaine, and (6) references in Table S1 in the Supplementary Material (*Optical imaging modalities used to study the neurovascular changes induced by cocaine*).

For instance, fiber photometry and microscopy using gradient refractive index (GRIN) lenses have been utilized for tracking neuronal activities in deeper brain regions through the insertion of optical fibers or GRIN lenses into the brain. Activation or suppression of specific types of neurons was reported via temporal recordings of ${\mathrm{Ca}}^{2+}$ signal changes from various calcium indicators.^12^^,^^13^^,^^101^^–^^106^ Compared with MIP, these two technologies are advanced in deeper brain accessibility and measurements from freely moving animals. However, photometry is a point detection of cell ensembles near the optical fiber tip without cellular resolution. Although microscopy with GRIN lenses can track neuronal activities with cellular resolution, its FOV is limited (<0.5 to 1 mm),^107^ which results in challenges in capturing the synchronization/interaction between neurovascular networks across a wide FOV. In addition, these modalities require a more invasive procedure, i.e., insertion of an optical fiber or a rod lens into the deeper brain, which can potentially induce tissue damage in the surrounding area of the implantation and confound experimental results.

Two-photon microscopy has advanced to image cellular and microvascular function in the cortex. Now, it has been increasingly used for imaging neuronal^108^^,^^109^ and vascular^110^ dynamics in response to cocaine using specific fluorescent indicators. Although it allows 3D reconstruction of neuronal or microvascular morphology and capturing cellular activities using fluorescent ${\mathrm{Ca}}^{2+}$ indicators for functional imaging studies, its FOV is limited and its temporal resolution is lower than that of MIP (i.e., 2–3D laser scanning versus a full-field imaging) for capturing rapid changes in large-scale neurovascular networks. Unlike confocal or two-photon microscopy for flow imaging, ODT measures CBF velocity based on the intrinsic Doppler effect of moving red blood cells (RBCs). Therefore, it does not require a fluorescent tracker, which is highly desirable for monitoring brain response to stimulants that could otherwise induce confounding artifacts or decay of image contrast over time. Advances of 1.3  μm μODT developed in our lab facilitate 3D imaging of CBF velocity networks across arteries, veins, and capillaries,^111^ including (1) quantitative 3D CBF velocity imaging, (2) high sensitivity (<20  μm/s), (3) label-free, (4) relatively large FOV (e.g., $3\times 2.4\times 1.5\;{\mathrm{mm}}^{3}$), and (5) high resolution (<6  μm). This technique has proven uniquely useful for detecting drug (e.g., cocaine) induced neurovascular disruption (e.g., microischemia, flow redistribution), as shown in Figs. 3, 4, and 5 above.

Besides ${\mathrm{Ca}}^{2+}$ fluorescence and vascular imaging, as discussed above, other optical imaging such as photoacoustic imaging demonstrated its capability to visualize hemodynamic changes in response to acute administration of cocaine.^112^^,^^113^ Although the imaging depth of this optical absorption imaging is advanced, it has a trade-off between imaging depth and spatial resolution. So far, there are limited applications in neuronal imaging for studying cocaine’s effects on the brain. Alternatively, the MIP system with its ability to simultaneously detect neuronal, astrocytic, and vascular activities confers an advantage by enabling both structural and functional imaging of the neurovascular system at high-spatiotemporal resolution over a larger FOV.

By contrast, other non-optical modalities such as PET and MRI provide noninvasive whole-brain imaging without depth limitation on rodent brains (Table S2 in the Supplementary Material). For example, microPET has been used to examine cocaine-induced effects on the metabolic activities of rodents.^114^^,^^115^ In addition, MRI with different contrast agents such as manganese (for mapping neuronal activity) and iron-oxide (for CBV) has been utilized to reveal brain region-specific changes with different parameters in response to acute or chronic cocaine exposure and abstinence.^4^^,^^116^^–^^120^ However, compared with optical imaging, their spatiotemporal resolution is relatively compromised, making it potentially challenging to distinguish cocaine’s effects on neurons versus the vascular effects of cocaine in the brain.

Nevertheless, each imaging modality offers distinct insights into cocaine-induced neurovascular dynamics with different advantages and disadvantages (Tables S1 and S2 in the Supplementary Material). Optical imaging techniques enable higher-resolution functional imaging but are often limited in FOV and penetration depth. By contrast, non-optical modalities offer superior depth/brain region coverage with various parameters but may lack the spatiotemporal resolution necessary to capture rapid neural activity at the cellular level. Given the complex and multifaceted nature of the neurovascular networks, multimodal approaches such as the MIP confer an advantage to provide a more comprehensive perspective on cocaine-induced changes with high-resolution measurements of neuro- and hemodynamics in rodent brains. Utilizing and advancing these integrative approaches will facilitate the discovery of new knowledge on neurochemical changes induced by addictive drugs, aid in identifying therapeutic targets, and help develop more effective clinical interventions.

## 8 Summary and Outlook

Optical imaging remains a crucial tool for studying the brain. Multichannel approaches have been extended to the microscopic system for detecting multiple aspects of brain physiology,^121^ as well as for imaging neurovascular activity in the NAV network.^47^ Its ability to provide high spatial and temporal resolution makes it ideal for capturing the intricate details of hemodynamic and neuronal responses, as well as the effects of cocaine on brain function. Previously, astrocytes were largely considered to only provide structural support and nutrients to surrounding neurons. However, recent research has significantly expanded the understanding of astrocytes, revealing their diverse roles in regulating the synaptic microenvironment and modulating CBF in response to neural activity through neurovascular coupling. Our findings indicate that acute cocaine induces widespread cerebral vasoconstriction and a reduction in CBF. Interestingly, chemogenetic inhibition of astrocyte activation was shown to eliminate cocaine-induced vasoconstriction, attenuate reductions in CBF, and limit neuronal calcium influx.^47^ These results suggest that astrocytes play a vital role in regulating both synaptic environment and vascular tone during cocaine responses. Consequently, targeting astrocytes may offer a promising therapeutic strategy for mitigating cocaine-induced neuronal toxicity and hypoxia.

With the increasing use of optical technologies to study awake animals, researchers can now investigate drug effects without the confounds from anesthesia.^78^^,^^122^ One major technical challenge in imaging awake animals, especially for capturing multiple components in brain tissue, is the presence of motion artifacts,^111^ which result from brain displacements during active behavior. A potential solution is to increase the image acquisition rate to minimize misalignment and deformation caused by movement. In our lab, the following strategies have been used to address this issue: (1) a multilayer cover glass was implanted to ensure that the gap between the removed skull and the brain was filled up, thereby immobilizing the brain tissue and minimizing motion artifacts during imaging; (2) animal training to habituate the animals to the imaging procedure, including (a) placing the animal on a custom air floating mobile cage and mounting the head-plate to a fixed-frame on the image stage to stabilize the relative position between the animal’s head and the imaging probe and (b) illuminating the animal brain through the cranial window using a time-sharing scheme to ensure multichannel light exposure to simulate the imaging condition. The detailed training protocol was provided previously.^78^

More recently, another effective approach involves applying artificial intelligence (AI), such as deep learning, to remove these motion artifacts^111^ or suppress the background noise,^123^ which has been initialized and applied in many laboratories.^124^^–^^127^ Currently, our lab uses self-supervised learning algorithms to denoise and minimize motion artifacts.^111^ Briefly, the model learned vascular features such as intensity and vessel connectivity from intensity remapping and vessel cropping modules. Then, a binarized mask segmenting the imaged vessels was created and applied to raw data to reduce background noise as well as stripe-like artifacts, allowing more accurate restoration of microvascular networks. These advancements will significantly enhance the capability of optical imaging to capture neurovascular dynamic changes and transient cellular activities, such as those in neurons and/or astrocytes, etc., in awake animal models.

In addition to examining neurovascular changes via optical imaging modalities, researchers have explored brain-computer interfaces (BCIs) as a means to modulate addictive behavior. Although they vary in design and purpose, they typically detect biomarkers associated with drug addiction and deliver targeted stimulations to achieve neuromodulation (summarized in Chen et al.^128^). For instance, a pilot study reported the clinical efficacy of deep-brain electrical stimulations targeting addiction-related brain regions in treating refractory cocaine dependence.^129^ Although they offer a potential treatment approach for substance use disorders (SUDs), further research using optical imaging remains essential to enhance our understanding of the complexity of SUDs and integrate this knowledge to enhance the effectiveness of BCI-based interventions.

It is clear that optical imaging tools are growing in importance for examining the impact of drugs on cerebral vasculature, neuronal, and astrocytic activities. Furthermore, combining these tools with transgenic mice and GECIs provides a powerful approach to selectively observe the response of specific cell types to various perturbations, as demonstrated with cocaine in this study. An exciting frontier is the role of astrocytes in mediating cocaine’s effects in cerebral vasculature and determining if modulating astrocyte function may decrease the negative consequences of cocaine abuse in the vasculature.

## Supplementary Material

10.1117/1.NPh.12.S1. S14611.s01

## Data Availability

Data sharing is not applicable to this article because it is a review article.
