# Supplementary material for: Review of cocaine-induced brain vascular and cellular function changes measured in vivo with optical imaging
Source: Neurophotonics. 2025 May 28;12(Suppl 1):S14611. doi: 10.1117/1.NPh.12.S1.S14611 (PMC12118881; doi:10.1117/1.NPh.12.S1.S14611)
Supplement: Supplementary file 1 [file NPh_012_S14611_SD001.docx]

**Supplemental Materials**

Manuscript for **Neurophotonics**: UNDERSTANDING BRAIN DISEASE WITH LIVE IMAGING

**Review of Cocaine-induced Brain Vascular and Cellular Function Changes Measured *in vivo* with Optical Imaging**

C. Du^1^, H. Jeong^1^, A.P. Koretsky^2^, and Y. Pan^1^

**^1^** Department of Biomedical Engineering, Stony Brook University, Stony Brook, NY 11794, USA

**^2^** National Institute of Neurological Disorders and Stroke, National Institutes of Health, Bethesda,

MD 20892, USA

**Supplementary materials: Summary of optical and non-optical modalities commonly used in cocaine studies.**

**Supplementary Table 1. Optical imaging modalities used to study the neurovascular changes induced by cocaine**

|  | Classification | Labeling | Measurements | Cocaine dose | Effects of cocaine | Ref. |
| --- | --- | --- | --- | --- | --- | --- |
| Ca^2+^ detection | Fiber Photometry | - AAVdj-CaMKIIα-GCaMP6f-WPRE (for pan-neuronal) - Cre-dependent AAVdj-EF1α-DIO-GCaMP6f-WPR (for MSNs) | Ca^2+^ signals of NAc and MSNs.   - % ΔF/F - Firing frequency | Acute and chronic (10 mg/kg, i.p. for 7 days) | - Frequency of Ca^2+^ transients in NAc ↓ - Dysfunctional D1 MSNs when chronically exposed. | 101 |
|  | Fiber Photometry | - DAT-Cre- or Sert-Cre- dependent AAV-EF1α-DIO-GCaMP6m for dopamine and serotonin neurons) | Ca^2+^ signals of VTA dopamine and DRN serotonin neurons.   - % ΔF/F - Initiation and rise/decay time | 0.25, 0.50, 1.00 mg/kg/infusion, i.v. | - Dopamine and serotonin neurons ↓. | 103 |
|  | Fiber Photometry with EEG | - AAV5-MCHp-GCaMP6f | Ca^2+^ signals of lateral hypothalamus MCH neurons and EEG (0.5-9Hz)   - % ΔF/F - EEG delta and theta ratio | SA 0.75mg/kg/  infusion, i.v. | - Disrupted EEG waveform and MCH neuronal activities. | 105 |
| Ca^2+^ imaging | Microscope with GRIN lens | - Rhod2/AM in D1- and D2-EGFP BAC transgenic mice | Ca^2+^ signals of cortical and striatal D1- or D2-MSNs   - % ΔF/F and slope (%/min) | 8mg/kg, i.p. | - D1-MSNs ↑. - D2-MSNs ↓. | 12* |
|  | Microscope with GRIN lens | - Rhod2/AM in D1- and D2-EGFP BAC transgenic mice | Ca^2+^ signals of striatal D1- or D2-MSNs   - % ΔF/F and slope (%/min) - Response latency (min) | Acute (8mg/kg) and chronic (30mg/kg, i.p.) | - Dopamine signaling ↓ by chronic cocaine. | 13* |
|  | Miniaturized Microscope with GRIN lens | - AAV1-CAG-Flex-GCaMP6s-WPRE.SV40 | Ca^2+^ signals of D1- or D2-MSN clusters in dorsal striatum.   - % ΔF/F - Synchrony and cell distance | 20mg/kg, i.p. | - D1-MSN cluster activity near DLS ↑. - D2-MSN near DMS ↓. | 102 |
|  | Miniaturized Microscope with GRIN lens | - Cre-dependent AAV2/5-CAG-DIO-GCamp6f-WPRE-SV40 | Ca^2+^ signals of IL-NAc   - Z-scored activity - Response latency (s) | SA 0.33mg/  0.2ml/infusion, i.v. | - IL-NAc activity ↓ before the cocaine-seeking action but disrupted by abstinence. | 104 |
|  | Miniaturized Microscope with GRIN lens | - Sodium fluorescein (Na-F) | Fluorescence intensity in PFC   - Na-F extravasation | 5-20mg/kg, i.p. | - Dose-dependent Na-F extravasation ↑ acutely. | 106 |
|  | Two-photon Microscopy | - FITC-conjugated dextran-2000 | Fluorescent signal from vasculature at 50-150µm depth   - Cerebral blood flux (pL/s) - Vascular pulsatility (µm ms) | 15mg/kg, i.p. for 5 days | - Flux and pulsatility ↓ in penetrating and surface arteries, and ascending veins | 110 |
|  | Two-photon Microscopy | - AAV2/9-CaMKIIα-GCaMP6s-WPRE-pA (for excitatory neurons) - vGAT-Cre-dependent AAV2/9-Syn-Flex-GCaMP6s-WPRE-SV40 (for GABAergic neurons) | Ca^2+^ signals from neurons at 100-200µm depth.   - Firing frequency - % of silent vs active cells after cocaine injection | 10mg/kg, i.p. | - Excitatory FrA neurons ↓ - No significant changes for GABAergic neurons. | 108 |
|  | Two-photon Microscopy | - AAV1-Syn-flex-GCaMP6f-WPRE-SV40 | Ca^2+^ signals from cortical L2/3 pyramidal neurons.   - Activity rate (Hz) | 20mg/kg, i.p. | - Spontaneous and tactile-evoked activities of L2/3 pyramidal neurons ↓. | 109 |
| Optical absorption imaging | Photoacoustic Imaging | N/A | Cortical hemodynamics   - % Δphotoacoustic signal | 2.5, 5.0mg/kg, i.v. | - Dose-dependent blood volume ↑ | 113 |
|  | Photoacoustic Imaging | N/A | Cortical hemodynamics   - HbO_2_, HbR, and sO_2_ - Spatiotemporal relative HbT | 5mg/kg, i.v. | - sO2 ↓. | 112 |

Blue indicates deeper brain imaging, while the modalities in red imaged cortices.

Asterisks (*) indicate papers published from our lab.

Abbreviations: NAc, nucleus accumbens; MSN, medium spiny neuron; VTA, ventral tegmental area; DRN, dorsal raphe nucleus; EEG, electroencephalogram; MCH, melanin-concentrating hormone; SA, self-administration; GRIN, gradient refractive index; EFGP, enhanced green fluorescent protein; BAC, bacterial artificial chromosome; DLS, dorsolateral striatum; DMS, dorsomedial striatum; IL, infralimbic cortex; PFC, prefrontal cortex; FITC, fluorescein isothiocyanate; FrA, frontal association cortex; HbO_2_, oxygenated hemoglobin; HbR, deoxygenated hemoglobin; sO2, changes in blood oxygenation; HbT, total hemoglobin;

**Supplementary table 2. Preclinical non-optical imaging modalities used to study the neurovascular changes induced by cocaine**

|  | Classification | Labeling | Measurements | Cocaine dose | Effects of cocaine | Ref. |
| --- | --- | --- | --- | --- | --- | --- |
| Non-optical imaging | Manganese-enhanced MRI | - MnCl_2_-4H_2_O (for Mn^2+^ as a Ca^2+^ analog into activated neurons) | - % MRI signal - Anatomical activation maps | 0.5, 2.0mg/kg, i.v. | - Region-specific neuronal activity ↑. | 4 |
|  | Manganese-enhanced MRI | - MnCl_2_-2H_2_O | - ΔT1 - Anatomical activation maps | SA 0.8mg/kg/  infusion, i.v. | - Neuronal activity ↓ by abstinence. | 116 |
|  | BOLD-fMRI | - N/A | - % BOLD signal - Anatomical activation maps | Acute and chronic (15mg/kg, i.p. for 7 days) | - Region-specific BOLD signals ↑ in acute cocaine. - ↓ in chronic cocaine. | 117 |
|  | Multimodal fMRI | - N/A | - % BOLD, CBF, and CMRO_2_ - Anatomical activation maps | 1mg/kg, i.v. | - CBF, CMRO_2_ ↑ - Region-dependent changes in BOLD signals. | 120 |
|  | fMRI | - P904 (for CBV weighted contrast) | - % fMRI signal (%CBV) - Peak amplitude, time to peak | 30mg/kg, i.p. | - Region specific %CBV ↓ in dopamine transporter KO mice. | 119 |
|  | fMRI | - Iron-oxide contrast agent (for CBV weighted contrast) | - % fMRI signal (%CBV) - Anatomical activation maps | SA 0.75mg/kg  /infusion, i.v. | - %CBV to acute cocaine ↓ by chronic exposure followed by abstinence. | 118 |
|  | microPET | - 11C-labeled-cocaine | - Pharmacokinetics of 11C-cocaine | 0.00039mg/kg of 903 µCi | - Faster clearance of cocaine and highly correlated CBV changes under isoflurane than α-chloralose | 122* |
|  | microPET | - 18F-fluorodeoxyglugose (FDG) | - SUV - Metabolic maps | SA 0.8mg/kg  /infusion, i.v. | - Glucose uptake in specific cortical areas of addict-like rats ↓. - Striatal and prefrontal metabolic activities ↓. | 114 |
|  | microPET | - ^18^F-3-fluoro-5-[(pyridine-3-yl)ethynyl]benzonitrile (FPEB) | - ΔBP_ND_ - Statistical parametric maps | SA 0.3mg/kg/  infusion, i.v. | - Glutamate, mGluR5 availability ↓. | 115 |

Asterisks (*) indicate papers published from our lab.

Abbreviations: fMRI, functional magnetic resonance imaging; BOLD, blood-oxygen-level-dependent; CBV, cerebral blood volume; CBF, cerebral blood flow; CMRO_2_, cerebral metabolic rate of oxygen; PET, positron emission tomography; BP_ND_, nondisplaceable binding; mGluR5, metabotropic glutamate receptor type 5; SUV, standardized uptake value
